# Hypermethylation in the *ZBTB20* gene is associated with major depressive disorder

**DOI:** 10.1186/gb-2014-15-4-r56

**Published:** 2014-04-02

**Authors:** Matthew N Davies, Lutz Krause, Jordana T Bell, Fei Gao, Kirsten J Ward, Honglong Wu, Hanlin Lu, Yuan Liu, Pei-Chein Tsai, David A Collier, Therese Murphy, Emma Dempster, Jonathan Mill, Alexis Battle, Sara Mostafavi, Xiaowei Zhu, Anjali Henders, Enda Byrne, Naomi R Wray, Nicholas G Martin, Tim D Spector, Jun Wang

**Affiliations:** 1 Department of Twin Research & Genetic Epidemiology, King’s College London, St Thomas’ Hospital Campus, Westminster Bridge Road, London SE1 7EH, UK; 2 Queensland Institute of Medical Research, Brisbane, QLD 4006, Australia; 3 BGI-Shenzhen, Shenzhen 518083, China; 4 SGDP Centre, Institute of Psychiatry, King’s College London, London SE5 8AF, UK; 5 Medical School, University of Exeter, Exeter EX1 2LU, UK; 6 Queensland Brain Institute, University of Queensland, St Lucia, QLD 4072, Australia; 7 Stanford University, Stanford, CA 94305, USA

## Abstract

**Background:**

Although genetic variation is believed to contribute to an individual’s susceptibility to major depressive disorder, genome-wide association studies have not yet identified associations that could explain the full etiology of the disease. Epigenetics is increasingly believed to play a major role in the development of common clinical phenotypes, including major depressive disorder.

**Results:**

Genome-wide MeDIP-Sequencing was carried out on a total of 50 monozygotic twin pairs from the UK and Australia that are discordant for depression. We show that major depressive disorder is associated with significant hypermethylation within the coding region of *ZBTB20*, and is replicated in an independent cohort of 356 unrelated case-control individuals. The twins with major depressive disorder also show increased global variation in methylation in comparison with their unaffected co-twins. *ZBTB20* plays an essential role in the specification of the Cornu Ammonis-1 field identity in the developing hippocampus, a region previously implicated in the development of major depressive disorder.

**Conclusions:**

Our results suggest that aberrant methylation profiles affecting the hippocampus are associated with major depressive disorder and show the potential of the epigenetic twin model in neuro-psychiatric disease.

## Background

During development, dynamic changes to the epigenome play a critical role in establishing and maintaining each tissue within the body [1,2]. In particular, DNA methylation has been shown to play a critical role in the development of sub-regions of the brain. Epigenetic processes control several neurobiological and cognitive processes, including neurogenesis, the limbic system, neuronal activity, learning and memory, drug addiction, neurodegeneration and circadian rhythm [3]. Mutations in the methyl CpG binding protein 2 gene (*MECP2*) have been shown to lead to neurodevelopmental deficits, such as those associated with Rett syndrome [4], and aberrant DNA methylation signatures have been observed in several neuropsychiatric disorders, including schizophrenia and bipolar disorder [5]. There is considerable interest, therefore, in investigating the role of epigenetics in the development of other psychiatric diseases, such as major depressive disorder (MDD) [3,6-10].

Although genetic variation and environmental stressors are believed to increase an individual’s susceptibility to MDD, genome-wide association studies have not yet identified any replicated associations with depression that could explain the full etiology of the disease [3,11]. Twin studies of MDD have estimated its heritability to be approximately 37% [12], but may be higher for recurrent and early onset MDD [13]. However, the majority of monozygotic twin (MZT) pairs are discordant for MDD (only 20% of male and 38% of female MZT pairs show concordance for the disorder under the DSM-IV criteria [14]).

Several lines of evidence suggest a role for epigenetic factors in the development of depression. The delayed onset of the condition along with its episodic nature strongly suggests that it may have an epigenetic component [3]. Several studies of animal models for depression indicate that epigenetic processes may play an essential role in the pathology of the disease. In particular, several mice studies showed that the antidepressants imipramine, tranylcypromine and fluoxetine were able to induce epigenetic changes [15]. A human study comparing 39 unrelated, postmortem frontal cortex MDD samples to 26 controls [16] identified several differentially methylated regions enriched for neuronal growth and developmental genes, although these failed to replicate. Uddin *et al.*[17] compared blood methylation profiles of 33 subjects with a lifetime history of depression and 67 non-depressed adults using the 27k array and demonstrated that genome-wide methylation profiles distinguish between depressed and non-depressed individuals. It has been suggested that childhood adversities could increase depression risk via epigenetic mechanisms [6,18-22]. There is also increasing evidence to suggest that epigenetic variation between MZT pairs may play a key role in the etiology of psychopathology and contribute to phenotypic disconcordance [23].

**Figure 1:**
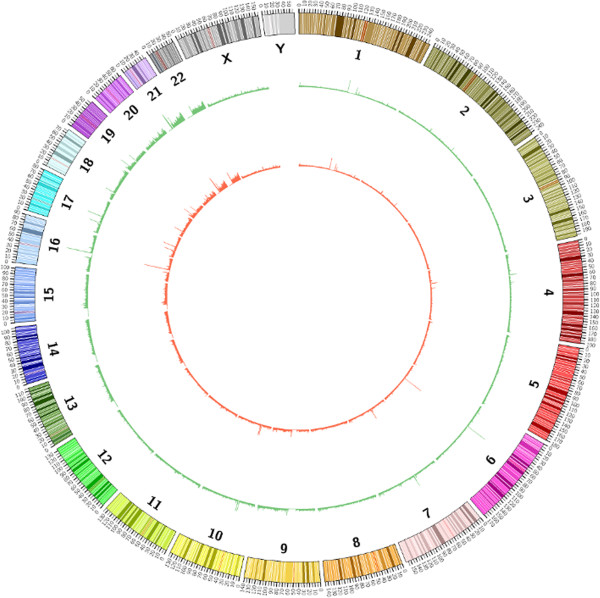
**Genomic methylation profiles of a female monozygotic twin pair discordant for major depressive disorder (MeDIP-seq data of depressed and non-depressed twin shown in red and green, respectively).** Although the overall patterns are extremely similar, differential methylation does occur at specific loci.

In this study we used methylated DNA immunoprecipitation combined with ultra-deep sequencing (MeDIP-seq) to provide comprehensive coverage of the methylomic landscape in order to compare blood samples between MZT pairs discordant for MDD in two independent datasets (Figure 1). The first cohort (UK) comprised 27 discordant twin pairs from the UK while the second cohort (Australia) comprised 23 discordant pairs from Australia. Discordant MZT pairs constitute a powerful design for epigenetic studies, as the genomic DNA sequence is identical within twin pairs and SNPs and other DNA sequence variations are not confounding factors. Additionally, twin pairs are generally exposed to similar environmental influences and important age and cohort effects are controlled within the paired comparison.

## Results and discussion

**Figure 2:**
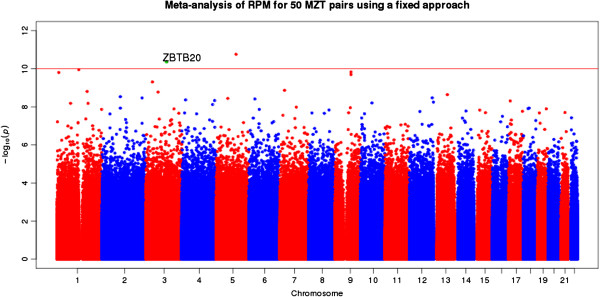
**Manhattan plot showing the *P-*** **values of approximately 10 M 500 bp bins from the meta analysis with the *ZBTB20 *** **DMR identified in the plot.**

**Figure 3:**
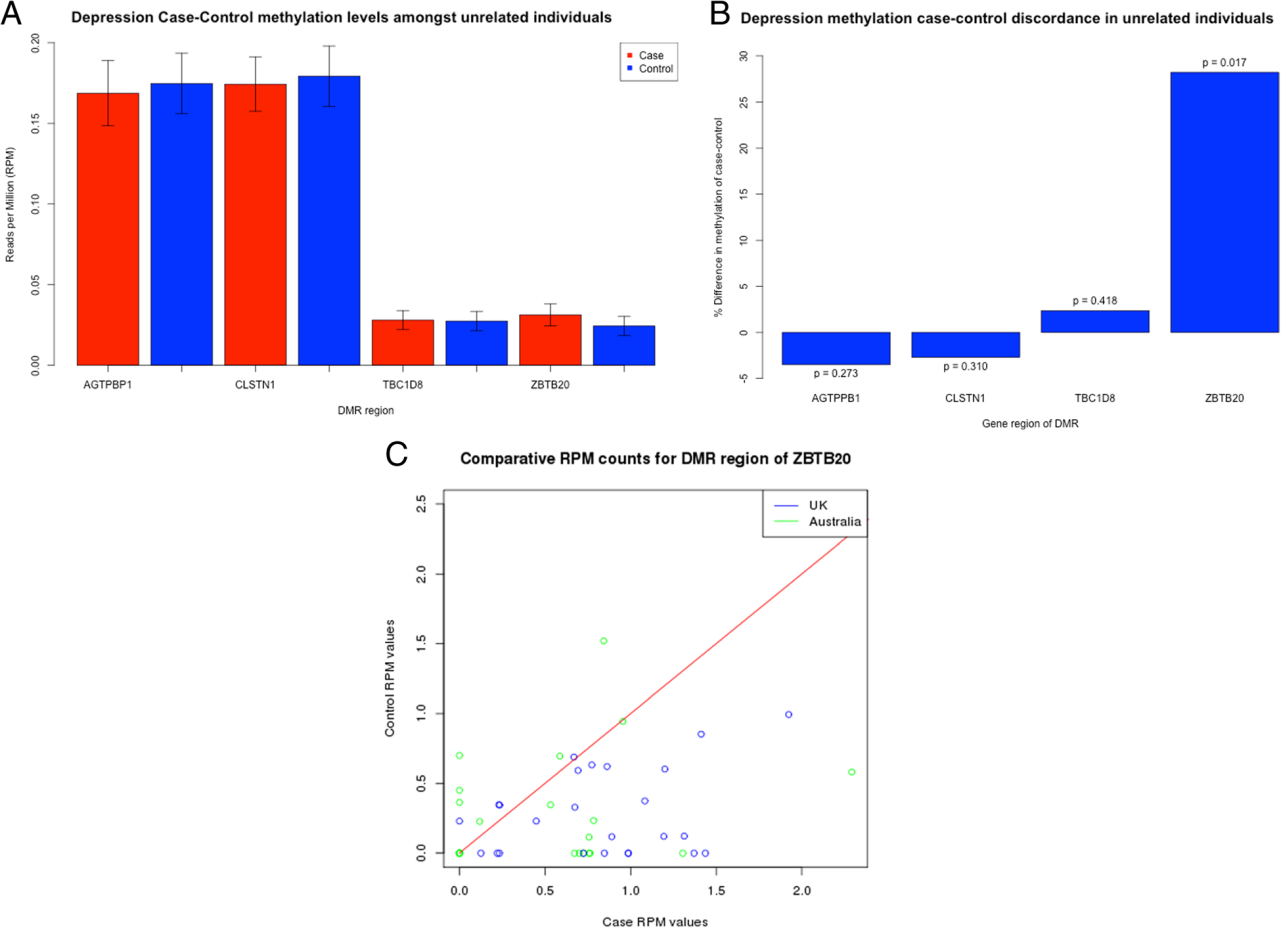
**(A,B) Replication of differentially methylated genes in an independent case-control cohort of 118 depressed and 236 control unrelated females.** The plot shows significantly increased methylation levels of the *ZBTB20* DMR region in individuals with MDD. **(C)** Case versus control RPM counts for the DMR of *ZBTB20* in 50 MZ twin pairs discordant for MDD.

The regression analysis of the 27 UK discordant MZT pairs alone did not clearly identify any differentially methylated region (DMR) of genome-wide significance (using a conservative significance level of 9 × 10^-10^; Table S1 in Additional file 1). However, several of the most differentially methylated genes were related to the pathology of MDD, namely *CADPS1*, *PTPRM* and *ZBTB20* (zinc finger and BTB domain containing 20 gene). Similarly, the regression analysis of 23 Australian discordant MZT pairs did not identify any DMR of genome-wide significance (Table S4 in Additional file 1). The second most differentially methylated gene (*EPHB1*), however, was related to the etiology of MDD (Additional file 1). The meta-analysis of both UK and Australian datasets identified 17 DMRs of genome-wide significance (*P* < 0.05, Bonferoni adjusted for approximately 11 M tests; Table 1; Figure 2). Four of these 17 DMRs were located within genes related to the pathology of MDD, namely *ZBTB20*, *AGTPBP1*, *TBC1D8* and *CLSTN1*, and selected for replication. The region was replicated in an independent replication cohort of 354 unrelated, age-matched females and showed an increased methylation of 28.2% in the 118 MDD cases compared to the 236 controls (*P* = 0.018, *t*-test; Figure 3a,b). A linear regression model of the *ZBTB20* region run on the independent case-control samples adjusting for age, body mass index (BMI) and smoking status retained significance (*P* = 0.0487). *ZBTB20* contains the second most significantly differentially methylated region identified in the meta-analysis, with an increased methylation level in cases (*P* = 0.00048, Bonferoni adjusted for approximately 11 M tests). The gene plays an essential role in the specification of the Cornu Ammonis-1 (CA1) field identity in the developing hippocampus. The RPM (reads per millions) value of the DMR is consistently higher in the depressed cohort in relation to the control (Figure 3c).

**Table 1:** Meta-analysis of 54 UK and 46 Australian blood samples using a fixed effects model approach

**Chromosome**	**Start**	**Stop**	***P-*** **value**	**Adjusted *P-*** **value**	**Gene start**	**Gene stop**	**Gene name**	**Distance**	**Description**
Chr5	63738001	63738501	1.75E-11	**0.00019**	63802451	63908121	*RGS7BP*	63950	Regulator of G-protein signaling 7 binding protein
Chr3	114618751	114619251	4.34E-11	**0.00048**	**114056946**	**114866127**	***ZBTB20***	**-**	**Zinc finger and BTB domain containing 20**
Chr1	120633001	120633501	1.12E-10	**0.00125**	120454175	120612317	*NOTCH2*	20684	Notch homolog 2 (Drosophila)
Chr9	84715501	84716001	1.47E-10	**0.00164**	84603686	84610171	*FLJ46321*	105330	FAM75-like protein FLJ46321
Chr1	9845751	9846251	1.58E-10	**0.00176**	**9789078**	**9884550**	***CLSTN1***	-	**Calsyntenin 1**
Chr9	88306501	88307001	2.02E-10	**0.00225**	**88161453**	**88356944**	***AGTPBP1***	-	**ATP/GTP binding protein 1**
Chr3	36140001	36140501	4.93E-10	**0.00549**	36422096	36589496	*STAC*	281595	SH3 and cysteine rich domain
Chr7	17309251	17309751	1.35E-09	0.01503	17338275	17385775	*AHR*	28524	Aryl hydrocarbon receptor
Chr1	166550001	166550501	1.56E-09	0.01737	166573152	166594473	*FMO9P*	22651	Flavin containing monooxygenase 9 pseudogene
Chr3	66970001	66970501	1.69E-09	0.01881	67048726	67061632	*KBTBD8*	78225	Kelch repeat and BTB (POZ) domain
Chr12	132823251	132823751	2.30E-09	0.02560	**132680916**	**132905905**	***GALNT9***	-	**UDP-N-acetyl-alpha-D-galactosamine**
Chr2	101764501	101765001	2.91E-09	0.03239	**101623689**	**101767846**	***TBC1D8***	-	**TBC1 domain family, member 8**
Chr12	57688251	57688751	3.37E-09	0.03752	**57647547**	**57704246**	***R3HDM2***	-	**R3H domain containing 2**
Chr2	221655001	221655501	3.42E-09	0.03807	222282746	222437010	*EPHA4*	627245	EPH receptor A4
Chr4	182369251	182369751	3.63E-09	0.04041	181985242	182080302	*LINC00290*	288949	Long intergenic non-protein coding RNA 290
Chr6	24236501	24237001	3.88E-09	0.04319	**24171982**	**24358280**	***DCDC2***	-	**Double cortin domain containing 2**
Chr4	15620251	15620751	4.30E-09	0.04787	**15606006**	**15657035**	***FBXL5***	-	**F-box and leucine-rich repeat protein 5**
Chr4	170932501	170933001	4.65E-09	0.05177	**170907747**	**170947429**	***MFAP3L***	-	**Microfibrillar-associated protein 3-like**
Chr16	55912001	55912501	4.93E-09	0.05488	**55880065**	**55989943**	***CES5A***	-	**Carboxylesterase 5A**
Chr12	126885001	126885501	5.67E-09	0.06312	126927026	126957331	*LOC100128554*	41525	Uncharacterized LOC100128554
Chr10	22007501	22008001	6.22E-09	0.06924	**21823100**	**22032559**	***MLLT10***	-	**Myeloid/lymphoid leukemia**
Chr1	172293751	172294251	6.57E-09	0.07314	**171810620**	**172381857**	***DNM3***	-	**Dynamin 3**
Chr1	75392751	75393251	6.61E-09	0.07358	75198861	75232360	*TYW3*	160391	tRNA-yW synthesizing protein 3

The nearest gene feature to a DMR is shown, DMRs occurring within a coding region are shown in bold. Bonferoni adjusted *P-*values with a significance <0.01 are also shown in bold.

### Observed methylation changes do not relate to anti-depressant use

The identified DMRs associated with MDD could reflect the consequence rather than the cause of the disorder. One possible consequence is use of anti-depressant medication, which was assessed as a confounder for the UK MZT pairs, for whom a record of drug usage was available. Two additional linear mixed models were calculated. The first included those individuals taking anti-depressant medication as an additional factor, the second eliminated twin pairs where the depressed twin was taking medication. Both produced results relatively consistent with the UK linear mixed model, suggesting that the observed methylation differences are not caused by anti-depressant medication based on data available to us (Tables S1, S2 and S3 in Additional file 1). *ZBTB20* is significantly associated with MDD to an unadjusted *P*-value of 2.99 × 10^-7^ if anti-depressant medication is included in the regression model and significant to a *P-*value of 1.28 × 10^-5^ even if all depressed individuals taking medication are removed from the study. The β coefficient of the DMR also remains consistent with the full linear model giving a β value of 1.073. Two other linear models, one where the anti-depressant medication was included as a co-factor and one where the model removed medicated twin pairs completely, produced β coefficients of 1.078 and 1.082, respectively. No association was observed between cell count and calculated methylation levels for the most significant DMRs in the UK linear mixed model (see Materials and methods).

Our MeDIP-seq data indicated that twins with MDD had a significantly increased variance in methylation when compared with their unaffected co-twin. Comparison of variance in global methylation between the depressed twin and their unaffected co-twin revealed a highly significant increased genome-wide variance in twins with MDD in both the UK and Australian cohorts (*P* < 2.2 × 10^-16^ in both datasets). This is in agreement with Byrne *et al.*[24], who used the much sparser 450 k array but also reported increased variance of methylation in the affected twin in a cohort of 12 MZT pairs, a subsample of the current Australian cohort. Byrne *et al.* failed to find any genome-wide significant DMRs in the 12 MZT pairs discordant for MDD. This could be the result of a limited sample size and/or the usage of the 450 k array, which has a much lower resolution than MeDIP-seq [25].

Several mouse studies have already demonstrated the importance of *ZBTB20* for normal hippocampal function. ZTBT20 targets hippocampal neurons as well as cerebellum granule cells [26], consistent with our observation of a high *ZTBT20* expression in the hippocampal, cerebellum and white matter regions of the brain. Conditionally deleting *ZBTB20* specifically in mature CA1 pyramidal neurons impairs long-term potentiation and NMDA receptor (NMDAR)-mediated excitatory post-synaptic currents [27]. *ZBTB20* is also crucial for the regionalization and volume of the archicortex [28], which plays a role in depression. In mice, mis-expression of *ZBTB20* causes the development of a compact homogenous pyramidal cell layer within the hippocampal region, which is linked to behavioural abnormalities [29].

Magnetic resonance imaging scans of MZT pairs discordant for MDD identified volume reduction in the left posterior hippocampal region in the depressed co-twin [30]. In MDD, the dentate gyrus, and pyramidal neuron soma size is significantly decreased [31-34], suggesting that altered neuronal development rather than outright neuronal loss is responsible for the structural abnormalities linked to depression [35]. This is consistent with the pattern of reduced hippocampal volume and impaired regionalization suggested by the mouse model. The DMR of *ZBTB20* we identified is hypermethylated in subjects with MDD and occurs within an identified splice region, which may have the effect of creating distinct isoforms based upon the specific methylation profile. *ZBTB20* is also functionally related to the only SNP so far associated with MDD to a genome-wide significance in a genome-wide association study (common SNP rs1545843 (minor allele frequency = 0.41)) [36] occurring within the gene *SLC6A15*, which like *ZTBT20* is associated with hippocampal structure. Down-regulation of *SLC6A15* causes a reduced hippocampal volume (an effect that was replicated in stress-susceptible mice) and lower *SLC6A15* expression in hippocampus reduces neural integrity and excitatory neurotransmission in the brain.

### *ZBTB20* shows specific gene expression in the hippocampus

**Figure 4:**
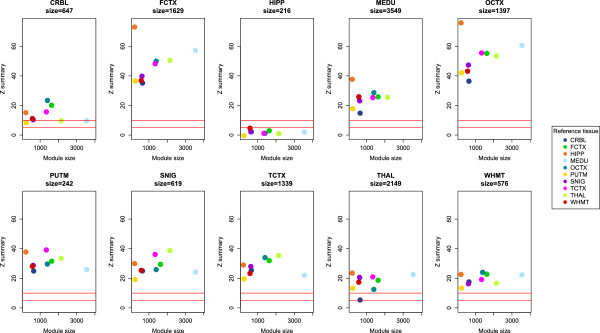
**Weighted gene co-expression networks using WGCNA showing unique co-expression of *ZBTB20 *** **in the hippocampus from a module containing 216 genes.**

Gene expression data of 932 brain samples from 10 different brain regions from 101 unrelated individuals taken from the Edinburgh Brain Bank (see Materials and methods) showed that, in the overall dataset, *ZBTB20* is highly expressed in the hippocampal, cerebellum and white matter regions of the brain and lowly expressed in the frontal, occipital and temporal cortex (Figure S1 in Additional file 1). A weighted gene co-expression network analysis using WGCNA [37] generated a hippocampus *ZBTB20*-cointaining module (with a total N = 216 genes) that was unique to the tissue (Z summary preservation statistic <10), suggesting that *ZBTB20* is co-expressed with a unique set of genes in the hippocampus, suggesting it has a key function in its regulation (Figure 4).

### RNA-seq data for MDD from NIMH Center for Collaborative Genomic Studies on Mental Disorders

Data were adjusted for the confounding covariates BMI, smoking, age, gender and various medication intake indicators. No association between total gene expression level of *ZBTB20* and MDD was observed (*P*-value >0.5). However, two non-standard exons identified within *ZBTB20* transcript variants (exon 33 in ENST00000463890 and exon 45 in ENST00000470556) are associated with unadjusted *P*-values of 0.041 and 0.04, respectively. For these two exons, we visually inspected the per-base read distribution in cases and controls, and observed a smooth shift of mean expression levels across all bases in each case (Figure S2 in Additional file 1). Both exons 33 and 45 are downstream of the DMR and are located at 114099729-114099787 and 114137901-114138014, respectively. In the mouse homologue gene, *Zbtb20*, two isoforms of the gene were shown to have distinct roles in the development of the hippocampus [29]. The expression of non-standard exons in *ZBTB20* may similarly have an impact on the development and regionalization of the human hippocampus.

### Limitations

Several possible limitations to our study should be noted. We used whole blood rather than brain samples of discordant MZT pairs to identify methylation differences associated with MDD, which is sub-optimal but clearly more accessible from living patients. Furthermore, if epigenetic studies are to be of clinical use, they will ultimately have to rely upon peripheral tissue biomarkers such as buccal, gut and white blood cells. However, a previous study comparing the methylation status of pre-mortem blood and post-mortem brain tissue [2] showed that significant variation in the methylation profile of brain tissue can be reflected in blood. Also, recent studies have shown that DMRs associated with both chronic pain [38] and ageing are similar in brain and blood tissue [39]. Although in the UK sample analyses of anti-depressant treatment could not explain the observed associations, other potential factors can not be entirely eliminated. For example, birth weight and chorionicity of the twin pair are possibly correlated with adult methylation levels [40,41], but that information was not available for the MZT pairs included in the study. Another potential confounding factor in the study was that the samples were predominately female. Our RNA-seq validation data set was more evenly distributed than the initial study with 30% of the subjects male (103 in case, 171 in control). It should, however, be noted that the RNA-seq association is only of nominal significance and would not retain significance if adjusted for multiple testing.

## Conclusions

Previous studies looking for genetic and epigenetic associations with MDD have largely been unsuccessful, possibly due to the complexity of the phenotype or the heterogeneity of the population. A meta-analysis of 50 pairs of MZ twins discordant for depression has identified a region of the genome consistently hypermethylated in the depressed cohort, a result that was replicated in an unrelated case-control population. Excitingly, the DMR occurs within the coding region of the *ZBTBT20* gene, which is associated with the structural integrity of the hippocampus. This supports current research regarding the etiology of MDD, which suggests it may be driven by a disorder of neuron structure [42-44]. Analysis of brain tissue and expression data in the region also supports a model whereby misexpression of *ZBTB20* may be associated with depression. This study represents the largest and most comprehensive study so far of genome-wide methylation differences in MZ pairs discordant for MDD and suggests that larger collaborative epigenetic twins studies are cost-effective and could provide even more clues to the etiology of complex traits.

## Materials and methods

The overall design was a meta-analysis of whole blood genome-wide methylation in two cohorts of MZT pairs discordant for MDD, followed by replication in an independent case-control group and exploration of expression and methylation signals in independent brain tissue samples.

### Included subjects

The 27 MZT pairs (n = 54) of the UK samples were selected from the TwinsUK Registry. The 54 participants were all females aged 23 to 73 years, of European ancestry and had no other psychiatric condition nor had they been diagnosed with any known neurodegenerative disorder. The study was approved by the St Thomas’ Hospital Research Ethics Committee (REC reference: EC04/015). All participants in the study provided written informed consent in accordance with the St Thomas’ Hospital Local Ethics Committee. UK twin pairs completed the Composite International Diagnostic Interview questionnaire [45]. A diagnosis of MDD was constructed from these questionnaires according to the DSM-IV criteria [46]. Whole blood samples were collected from the twins and stored at -80°C in EDTA tubes. DNA was extracted from 2 × 6 ml EDTA blood using the Nucleon Genomic DNA Extraction Kit BACC3 and stored at -20°C in TE Buffer.

The 23 MZT pairs of the Australian samples (n = 46) were drawn from the Australian Twin Registry. The 46 participants comprised 7 male and 16 female MZ twin pairs aged 25 to 73 years. Seven pairs were discordant for smoking and a partially overlapping set of seven twin pairs was discordant for alcohol dependence. The study was approved by the Human Research Ethics Committee of the Queensland Institute of Medical Research. The assessment of Australian MZT pairs included a diagnostic telephone interview, adapted from the Semi-Structured Assessment for the Genetics of Alcoholism (SSAGA) [47]. The SSAGA is a comprehensive psychiatric interview that was designed to assess life-time psychiatric disorders in adults according to DSM-III-R but subsequently updated to DSM-IV criteria and modified for use as a telephone survey instrument in Australia (SSAGA-OZ). SSAGA also assesses history of alcohol dependence and tobacco smoking with questions derived from the Composite International Diagnostic Interview [48]. Structured interviews were administered by trained telephone interviewers, closely supervised by a clinical psychologist. DNA was extracted from whole blood using a salt extraction method [49].

### Sample preparation for MeDIP-seq

All sample preparation and MeDIP-sequencing was performed by the BGI-Shenzhen, Shenzhen, China. Extracted DNA was fragmented using a Covaris sonication system and sequencing libraries were prepared from 5 μg fragmented genomic DNA. End repair, <A > base addition and adaptor ligation steps were performed using Illumina’s Single-End DNA Sample Prep kit. Adaptor-ligated DNA was immunoprecipitated by anti-5mC using a commercial antibody (Diagenode), and MeDIP products were validated by quantitative PCR. MeDIP DNA was purified with ZYMO DNA Clean & Concentrator-5 columns, and amplified using adaptor-mediated PCR. DNA fragments between 220 and 320 bp in size were gel-excised, and amplification quality and quantity were evaluated by Agilent BioAnalyzer analysis. The libraries were subjected to highly parallel 50 bp single-end sequencing on the Illumina HiSeq platform.

### Sequencing quality control and alignment

From the raw fastq files, Illumina quality scores were converted into Sanger Phred quality scores using MAQ [50]. Quality control was performed on the raw sequence data using in-house scripts and FastQC [51]. After stringent quality control, an average of 19 million uniquely mapped 50 bp reads were obtained from each of the 100 samples. Alignment to hg19 was performed using the Burrows-Wheeler algorithm [52]. The MEDIPS package [53] was used to calculate RPM scores by defining bin sizes of 500 bp with an overlap of 250 bp across the genome (Figure 1). The total number of 500 bp bins generated by the MEDIPS packages was 12,145,229. The number of bins was filtered to include only bins where more than 10% of samples had a read coverage greater than 0. For the UK samples, this reduced the number to 11,132,286 bins, for the Australian samples to 10,480,864. For both data sets, the raw FastQ files and calculated RPM scores from the aligned data are available from the Gene Expression Omnibus (GEO) database [54]. The UK samples are Study ID GSE54222 and the Australian samples are Study ID GSM1313979.

### Linear mixed effect models

A linear mixed effect model was fitted for the RPM values of each 500 bp bin using the R package lmer [55]. Models were derived separately for the UK and Australian cohorts, reflecting the different fixed and random effects that needed to be incorporated for the two datasets. For the UK dataset, the linear mixed effect model incorporated depression status, age, BMI, smoking and alcohol consumption as fixed effects predictors [40,56-58] and twin pair as random effect (Table S1 in Additional file 1). In order to determine whether the discordant methylation could be a result of anti-depressant medication, two further linear mixed models were calculated. The first included those individuals taking anti-depressant medication as an additional factor, the second eliminated twin pairs where the depressed twin was taking medication from the study (Tables S2 and S3 in Additional file 1). Research has suggested that heterogeneity in whole blood cell counts could confound estimates of DNA methylation levels [59,60]. Cell count data were available for 21 twin pairs in the UK dataset; the top hits from the linear mixed model were analysed for evidence of association with cell counts of lymphocytes, neutrophils, eosinophils, monocyte, and total white blood and blood cell counts. For both data sets, the variance in methylation for cases and controls was compared for each bin.

The linear mixed effect model for the Australian dataset also incorporated depression status, age, tobacco addiction (yes/no) and alcohol dependence (yes/no) as fixed effects and twin pair as a random effect; medication data were not available for these samples. Sex was also incorporated as a fixed effect, but BMI was excluded, as this information was unavailable for four twin pairs. We investigated the effect of BMI as a covariate by fitting a second linear mixed effect model for the subset of 19 Australian twin pairs with available BMI information. Incorporating BMI as a fixed effect had only a minor effect on the *P*-values observed for the DMRs shown in Table S4 in Additional file 1.

### Meta-analysis

As the UK and Australian datasets were drawn from different populations and modeled with different fixed effects, the data were integrated through a meta-analysis. A fixed effect inverse variance meta-analysis was carried out on all approximately 11 M bins of the UK and Australian datasets, using GWAMA [61]. *P*-values were Bonferoni adjusted to correct for multiple testing. We only present results for DMRs that show no strong evidence for heterogeneity in the meta-analysis as evaluated by the Cochran’s Q statistic (Cochran’s Q *P* > 0.05) [62] and the I^2^ statistic (I^2^ < 0.75) [63].

### Replication in independent case-control cohort of 354 unrelated females

Differentially methylated genes identified in the meta-analysis of UK and Australian MZT pairs were evaluated with the existing scientific literature to select for likely MDD-related DMRs. Four DMRs were located within genes related to the pathology of MDD and selected for replication (see Results). For an independent case-control replication we included blood MeDIP-seq data of 354 unrelated, age-matched females from the EpiTwin project, 118 suffering from MDD and 236 controls. MeDIP-seq data of 118 cases and 236 controls were compared by *t*-test (RPM values of each 500 bp bin).

### Postmortem brain expression data obtained from the UK Brain Expression Consortium

The four DMRs selected for replication were also compared with an independent expression dataset of 932 postmortem brain samples collected from the Edinburgh Brain Bank as part of the UK Brain Expression Consortium [64]. Expression data were generated with the Affymetrix GeneChip Human Exon 1.0 ST Array and the dataset contains 932 brain samples of 10 different brain regions obtained from 101 unrelated individuals (24 male and 77 female) aged from 16 to 83 years. The 10 brain regions are cerebellum, frontal cortex, hippocampus, medulla, occipital cortex, putamen, substantia niagra, temporal cortex, thalamus and intralobular white matter. The WGCNA R package was used to analyze the data for incidence of conserved co-expression gene networks [65].

### Case-control MDD RNA-seq data

The cohort is of European ancestry and contains 463 individuals with recurrent MDD and 459 controls. A detailed description of RNA-sequence and phenotype data for this cohort is provided elsewhere [66,67]. RNA-sequencing was performed using whole-blood, with an average yield of 70 million reads per individual (50 or 51 bp, single-ended). Reads were mapped to the NCBI v37 *H. sapiens* reference genome using TopHat [68].Gene expression data were directly obtained from the previous study on this cohort [66]. Gene-level expression was quantified using HTSeq [69]. Additionally, we used samtools mpileup to quantify reads at each exonic position within the gene [70]. Only uniquely aligned reads with base quality of at least 30 were used for quantification. We then aggregated reads within the start and end points of each distinct exon identified in ENSEMBL *Homo sapiens* gene annotation, and normalized by the total read depth of each library. Total gene expression and exon expression of *ZBTB20* were tested for association with MDD status. Associating testing followed the procedure reported previously for this cohort: a logistic regression likelihood ratio test was used to test for association between expression levels and MDD status while accounting for environmental, demographic, and medication intake covariates [66]. The list of covariates include age, gender, BMI, smoking status, cholesterol and blood pressure medication intake indicators. Genotype, raw RNA-seq, quantified expression, and covariate data are available by application through the NIMH Center for Collaborative Genomic Studies on Mental Disorders. Instructions for requesting access to data can be found at NIMH Repository and Genomics Resource [71] and inquiries should reference the 'Depression Genes and Networks study' (D Levinson, PI) [66].

## Abbreviations

BMI: body mass index; bp: base pair; CA1: Cornu Ammonis-1; DMR: differentially methylated region; DSM-IV: Diagnostic and Statistical Manual of Mental Disorders, Fourth Edition; MDD: major depressive disorder; MeDIP-seq: methylated DNA immunoprecipitation combined with ultra-deep sequencing; MZT: monozygotic twin; PCR: polymerase chain reaction; RPM: reads per millions; SNP: single nucleotide polymorphism; SSAGA: Semi-Structured Assessment for the Genetics of Alcoholism.

## Competing interests

The authors declare that they have no competing interests.

## Authors’ contributions

FG, HW, HL, YL and JW were responsible for the sequencing and quality control of the MeDIP-seq. JM, ED and TM provided provided additional validation data. AB, SM and XZ provided and analyzed the RNA-seq data. UK Brain Expression Consortium provided gene expression brain data. KJW, P-CT and DAC provided twin data for the UK cohort. AH, EB and NRW provided twin data for the Australian cohort. Analysis and meta-analysis of both datasets was carried out by MND, LK and JTB. MND, LK, JTB, TDS and NGM drafted the manuscript. All authors read and approved the final manuscript.

## Supplementary Material

Additional file 1: Table S1 linear mixed model on TwinsUK dataset RPM values showing values for age, depression, smoking, alcohol and BMI. The nearest gene feature to a DMR is shown; DMRs occurring within a coding region are shown in bold. **Table S2.** linear mixed model on RPM factoring values for age, depression, smoking, alcohol, BMI and anti-depressant medication. The nearest gene feature to a DMR is shown, DMRs occurring within a coding region are shown in bold. **Table S3.** linear mixed model on RPM factoring values for age, depression, smoking, alcohol and BMI removing twin pairs taking anti-depressant medication. The nearest gene feature to a DMR is shown; DMRs occurring within a coding region are shown in bold. **Table S4.** linear mixed model on Queensland dataset RPM values showing values for age, depression, smoking, alcohol and BMI. The nearest gene feature to a DMR is shown; DMRs occurring within a coding region are shown in bold. **Figure S1.** averaged expression values of the *ZBTB20* gene across 10 brain regions. **Figure S2. ***ZBTBT20* exon 33 and exon 45 case-control comparison for the RNA-seq expression data. Click here for file
