# Supplementary material for: Hypermethylation in the ZBTB20 gene is associated with major depressive disorder
Source: Genome Biol. 2014 Apr 2;15(4):R56. doi: 10.1186/gb-2014-15-4-r56 (PMC4072999; doi:10.1186/gb-2014-15-4-r56)
Supplement: Additional file 1: Table S1 — linear mixed model on TwinsUK dataset RPM values showing values for age, depression, smoking, alcohol and BMI. The nearest gene feature to a DMR is shown; DMRs occurring within a coding region are shown in bold. Table S2. linear mixed model on RPM factoring values for age, depression, smoking, alcohol, BMI and anti-depressant medication. The nearest gene feature to a DMR is shown, DMRs occurring within a coding region are shown in bold. Table S3. linear mixed model on RPM factoring values for age, depression, smoking, alcohol and BMI removing twin pairs taking anti-depressant medication. The nearest gene feature to a DMR is shown; DMRs occurring within a coding region are shown in bold. Table S4. linear mixed model on Queensland dataset RPM values showing values for age, depression, smoking, alcohol and BMI. The nearest gene feature to a DMR is shown; DMRs occurring within a coding region are shown in bold. Figure S1. averaged expression values of the ZBTB20 gene across 10 brain regions. Figure S2. ZBTBT20 exon 33 and exon 45 case-control comparison for the RNA-seq expression data. [file gb-2014-15-4-r56-S1.docx]

Supplementary Table 1: Linear mixed model on TwinsUK dataset RPM values showing β values for age, depression, smoking, alcohol and BMI. The nearest gene feature to a DMR is shown, DMRs occurring within a coding region are shown in bold.

| Chr | start | Stop | X.Intercept. | age | depression | smoking | alcohol | BMI | pvalue | Gene | Description |
| --- | --- | --- | --- | --- | --- | --- | --- | --- | --- | --- | --- |
| chr2 | 55887751 | 55888250 | -1.147 | -0.003 | 0.932 | 0.356 | 0.242 | 0.017 | 1.50E-08 | **PNPT1** | **polyribonucleotide nucleotidyltransferase** |
| chr6 | 139528751 | 139529250 | -1.115 | 0.010 | 0.999 | -0.252 | -0.115 | 0.008 | 1.79E-08 | HECA | headcase homolog (Drosophila) |
| chr6 | 18241001 | 18241500 | -0.185 | 0.003 | 0.998 | 0.292 | -0.022 | -0.021 | 2.60E-08 | **DEK** | **DEK oncogene** |
| chr2 | 53454751 | 53455250 | -0.432 | -0.003 | -1.303 | 0.089 | 0.272 | 0.035 | 3.29E-08 | LOC727915 | uncharacterized LOC727915 |
| chr15 | 87282501 | 87283000 | 0.018 | 0.005 | -1.056 | 0.266 | 0.085 | 0.001 | 7.15E-08 | **AGBL1** | **ATP/GTP binding protein** |
| chr18 | 7832001 | 7832500 | 1.162 | -0.021 | 1.188 | -0.580 | -0.206 | -0.004 | 7.42E-08 | **PTPRM** | **protein tyrosine phosphatase, type, M** |
| chr6 | 135940501 | 135941000 | -1.219 | 0.009 | 1.083 | 0.043 | -0.055 | 0.006 | 8.19E-08 | **C6orf217** | **chromosome 6 open reading frame 217** |
| chr4 | 38369501 | 38370000 | -0.551 | 0.014 | 1.177 | 0.211 | -0.228 | -0.028 | 9.80E-08 | TBC1D1 | TBC1 (tre-2/USP6, BUB2, cdc16) |
| chr2 | 48168501 | 48169000 | 0.856 | -0.005 | -0.984 | 0.351 | 0.671 | -0.025 | 1.09E-07 | FBXO11 | F-box protein 11 (FBXO11 |
| chr4 | 15620251 | 15620750 | 0.972 | 0.013 | 0.863 | -0.137 | -0.260 | -0.066 | 2.02E-07 | CC2D2A | coiled-coil and C2 domain |
| chr1 | 90696251 | 90696750 | 0.310 | -0.001 | 1.226 | 0.179 | -0.308 | -0.026 | 2.15E-07 | ZNF326 | zinc finger protein 326 |
| chr13 | 43750001 | 43750500 | -1.050 | 0.012 | 1.250 | -0.143 | -0.028 | -0.006 | 2.27E-07 | ENOX1 | ecto-NOX disulfide-thiol exchanger |
| chr2 | 12048001 | 12048500 | 0.012 | 0.018 | -1.154 | 0.158 | 0.212 | -0.025 | 2.66E-07 | MIR4262 | microRNA 4262 |
| chr2 | 57364001 | 57364500 | -0.240 | 0.005 | -1.009 | 0.544 | -0.089 | 0.008 | 2.79E-07 | CCDC85A | coiled-coil domain containing 85A |
| chr8 | 130549751 | 130550250 | -0.389 | 0.012 | 1.207 | 0.162 | -0.061 | -0.032 | 3.13E-07 | GSDMC | gasdermin C |
| chr3 | 114618751 | 114619250 | 0.228 | -0.004 | 1.073 | -0.612 | 0.097 | -0.009 | 3.43E-07 | **ZBTB20** | **zinc finger and BTB domain containing 20** |
| chr1 | 58906001 | 58906500 | 0.445 | -0.010 | 1.230 | -0.057 | 0.084 | -0.017 | 4.18E-07 | OMA1 | OMA1 homolog, zinc metallopeptidase |
| chr10 | 19516501 | 19517000 | -0.051 | 0.005 | 0.986 | -0.427 | 0.431 | -0.029 | 4.32E-07 | ARL5B | ADP-ribosylation factor-like 5B |
| chr3 | 62424751 | 62425250 | -1.107 | 0.000 | 0.987 | -0.539 | -0.500 | 0.043 | 4.82E-07 | **CADPS1** | **Ca++-dependent secretion activator 1** |
| chr1 | 24012751 | 24013250 | -0.165 | 0.004 | 1.134 | 0.073 | -0.239 | -0.017 | 5.06E-07 | RPL11 | ribosomal protein L11 (RPL11) |

Supplementary Table 2: Linear mixed model on RPM factoring β values for age, depression, smoking, alcohol, BMI and anti-depressant medication. The nearest gene feature to a DMR is shown, DMRs occurring within a coding region are shown in bold.

| **Chr** | **Start** | **Stop** | **age** | **depress** | **smoking** | **alcohol** | **BMI** | **Meds** | **p value** | **Gene** | **Description** |
| --- | --- | --- | --- | --- | --- | --- | --- | --- | --- | --- | --- |
| chr2 | 55887751 | 55888250 | -0.003 | 0.930 | 0.380 | 0.245 | 0.016 | -0.376 | 1.547E-08 | PNPT1 | **polyribonucleotide nucleotidyltransferase** |
| chr6 | 139528751 | 139529250 | 0.010 | 0.997 | -0.234 | -0.112 | 0.007 | -0.229 | 1.879E-08 | HECA | headcase homolog (Drosophila) |
| chr6 | 18241001 | 18241500 | 0.003 | 1.003 | 0.262 | -0.026 | -0.020 | 0.332 | 2.364E-08 | DEK | **DEK oncogene** |
| chr2 | 53454751 | 53455250 | -0.003 | -1.300 | 0.068 | 0.268 | 0.035 | 0.141 | 3.363E-08 | ASB3 | ankyrin repeat and SOCS box-containing 3 |
| chr18 | 7832001 | 7832500 | -0.020 | 1.194 | -0.639 | -0.212 | -0.001 | 0.482 | 5.099E-08 | PTPRM | **protein tyrosine phosphatase, receptor** |
| chr15 | 87282501 | 87283000 | 0.005 | -1.060 | 0.303 | 0.086 | -0.001 | -0.391 | 6.207E-08 | AGBL1 | **ATP/GTP binding protein-like 1** |
| chr4 | 38369501 | 38370000 | 0.013 | 1.169 | 0.276 | -0.229 | -0.030 | -0.606 | 7.348E-08 | TBC1D1 | TBC1 domain family |
| chr6 | 135940501 | 135941000 | 0.009 | 1.085 | 0.029 | -0.057 | 0.006 | 0.151 | 7.699E-08 | C6orf217 | **chromosome 6 open reading frame 217** |
| chr2 | 48168501 | 48169000 | -0.005 | -0.985 | 0.358 | 0.673 | -0.025 | -0.078 | 1.080E-07 | FBXO11 | F-box protein 11 (FBXO11), |
| chr1 | 90696251 | 90696750 | -0.001 | 1.223 | 0.197 | -0.305 | -0.027 | -0.118 | 2.195E-07 | ZNF326 | zinc finger protein 326 |
| chr13 | 43750001 | 43750500 | 0.012 | 1.250 | -0.147 | -0.029 | -0.006 | 0.027 | 2.267E-07 | ENOX1 | Ecto-NOX disulfide-thiol exchanger 1 (ENOX1), |
| chr2 | 57364001 | 57364500 | 0.005 | -1.011 | 0.564 | -0.086 | 0.007 | -0.225 | 2.585E-07 | CCDC85A | coiled-coil domain containing 85A |
| chr2 | 12048001 | 12048500 | 0.019 | -1.149 | 0.115 | 0.205 | -0.023 | 0.329 | 2.654E-07 | MIR4262 | microRNA 4262 (MIR4262), |
| chr13 | 82560751 | 82561250 | -0.013 | -1.191 | 0.563 | -0.001 | 0.020 | -0.750 | 2.722E-07 | SPRY2 | sprouty homolog 2 (Drosophila) |
| chr8 | 130549751 | 130550250 | 0.011 | 1.200 | 0.212 | -0.053 | -0.034 | -0.325 | 2.794E-07 | GSDMC | gasdermin C |
| chr4 | 15620251 | 15620750 | 0.012 | 0.852 | -0.073 | -0.245 | -0.066 | -0.803 | 2.970E-07 | FBXL5 | **F-box and leucine-rich repeat protein 5** |
| chr3 | 114618751 | 114619250 | -0.004 | 1.078 | -0.656 | 0.081 | -0.007 | 0.457 | 2.992E-07 | ZBTB20 | **zinc finger and BTB domain containing 20** |
| chr1 | 58906001 | 58906500 | -0.010 | 1.222 | 0.000 | 0.093 | -0.019 | -0.366 | 3.617E-07 | OMA1 | OMA1 homolog, zinc metallopeptidase |
| chr4 | 112260501 | 112261000 | 0.007 | -1.192 | 0.386 | -0.050 | -0.025 | -0.411 | 4.226E-07 | PITX2 | paired-like homeodomain 2 |
| chr3 | 62424751 | 62425250 | 0.000 | 0.985 | -0.523 | -0.497 | 0.042 | -0.140 | 4.952E-07 | CADPS1 | **Ca++-dependent secretion activator** |

Supplementary Table 3: Linear mixed model on RPM factoring β values for age, depression, smoking, alcohol and BMI removing twin pairs taking anti-depressant medication. The nearest gene feature to a DMR is shown, DMRs occurring within a coding region are shown in bold.

| **Chr** | **Start** | **Stop** | **age** | **depression** | **smoking** | **alcohol** | **BMI** | **p value** | **Gene** | **Description** |
| --- | --- | --- | --- | --- | --- | --- | --- | --- | --- | --- |
| chr4 | 78290251 | 78290750 | -0.008 | -1.510 | 0.302 | -0.198 | 0.022 | 6.10E-10 | CXCL13 | chemokine (C-X-C motif) ligand 13 |
| chr2 | 53454751 | 53455250 | -0.013 | -1.410 | 0.207 | 0.460 | 0.020 | 3.215E-09 | ASB3 | ankyrin repeat and SOCS box-containing 3 |
| chr6 | 135940501 | 135941000 | 0.010 | 1.102 | 0.064 | -0.325 | 0.008 | 7.234E-09 | **C6of217** | **chromosome 6 open reading frame 217** |
| chr4 | 169263501 | 169264000 | 0.018 | -1.111 | 0.339 | 0.212 | 0.032 | 2.962E-08 | DDX60L | DEAD (Asp-Glu-Ala-Asp) box polypeptide 60-like |
| chr12 | 39545251 | 39545750 | 0.013 | -0.981 | 0.708 | 0.166 | 0.010 | 3.9165E-08 | KIF21A | kinesin family member 21A (KIF21A) |
| chr8 | 135801001 | 135801500 | 0.009 | 1.368 | -0.090 | 0.251 | -0.022 | 5.521E-08 | MIR30B | microRNA 30b (MIR30B) |
| chr4 | 5372501 | 5373000 | -0.004 | 1.145 | -0.805 | 0.105 | 0.031 | 7.6524E-08 | **STK32B** | **serine/threonine kinase 32B** |
| chr2 | 57364001 | 57364500 | 0.005 | -1.084 | 0.506 | -0.124 | 0.010 | 8.6648E-08 | CCDC85A | coiled-coil domain containing 85A |
| chr18 | 7832001 | 7832500 | -0.022 | 1.245 | -0.690 | -0.079 | -0.010 | 9.7331E-08 | **PTPRM** | **protein tyrosine phosphatase, receptor type, M** |
| chr4 | 38369501 | 38370000 | 0.014 | 1.196 | 0.232 | -0.340 | -0.029 | 2.06855E-07 | TBC1D1 | TBC1 domain family, member 1 (TBC1D1) |
| chr15 | 54929501 | 54930000 | 0.004 | 1.255 | 0.269 | 0.353 | -0.029 | 2.98876E-07 | UNC13C | unc-13 homolog C (C. elegans) |
| chr12 | 5663001 | 5663500 | -0.003 | -1.121 | 0.114 | 0.618 | 0.006 | 3.36947E-07 | ANO2 | anoctamin 2 |
| chr10 | 42449501 | 42450000 | -0.005 | 1.280 | 0.022 | -0.055 | -0.045 | 3.6662E-07 | LOC441666 | zinc finger protein 91 pseudogene |
| chr1 | 121257001 | 121257500 | -0.006 | 1.049 | -0.106 | 0.134 | -0.025 | 4.02892E-07 | LOC647121 | embigin homolog (mouse) pseudogene |
| chr1 | 182990501 | 182991000 | -0.001 | -1.302 | -0.067 | 0.136 | 0.007 | 4.05204E-07 | LAMC1 | laminin, gamma 1 (formerly LAMB2) |
| chr18 | 11872501 | 11873000 | -0.004 | -0.918 | 0.334 | 0.136 | -0.017 | 4.24429E-07 | **GNAL** | **guanine nucleotide binding protein (G protein)** |
| chrX | 74720751 | 74721250 | -0.006 | 0.710 | 0.233 | -0.681 | -0.045 | 4.82299E-07 | **ZDHHC15** | **zinc finger, DHHC-type containing 15** |
| chr3 | 78295751 | 78296250 | -0.007 | -1.232 | 0.464 | 0.058 | 0.006 | 5.17313E-07 | ROBO1 | roundabout, axon guidance receptor, homolog 1 |
| chr1 | 155936001 | 155936500 | 0.023 | 0.911 | -0.760 | 0.133 | 0.029 | 5.8406E-07 | ARHGEF2 | Rho/Rac guanine nucleotide exchange factor (GEF) 2 |
| chr15 | 87282501 | 87283000 | 0.013 | -1.014 | 0.423 | 0.127 | 0.016 | 6.14394E-07 | **ABGL1** | **ATP/GTP binding protein-like 1** |

Supplementary Table 4: Linear mixed model on Queensland dataset RPM values showing β values for age, depression, smoking, alcohol and BMI. The nearest gene feature to a DMR is shown, DMRs occurring within a coding region are shown in bold.

| Chr | Start | Stop | depression | sex | age | alcohol | smoking | pvalue | Gene | Description |
| --- | --- | --- | --- | --- | --- | --- | --- | --- | --- | --- |
| chr5 | 41245251 | 41245750 | 1.385 | -0.209 | -0.010 | -0.488 | 0.445 | 6.08E-08 | **C6** | **complement component 6** |
| chr3 | 134801751 | 134802250 | -1.317 | 0.333 | 0.038 | 0.497 | -0.098 | 8.86E-08 | **EPHB1** | **EPH receptor B1 (EPHB1), mRNA.** |
| chr 4 | 151897751 | 151898250 | 1.358 | -0.044 | -0.030 | -0.861 | 0.450 | 2.12E-07 | **LRBA** | **LPS-responsive vesicle trafficking** |
| chr17 | 57718251 | 57718750 | 1.216 | 0.126 | -0.002 | -0.373 | 0.915 | 2.25E-07 | **CLTC** | **clathrin, heavy chain (Hc) (CLTC), mRNA.** |
| chr3 | 150794001 | 150794500 | -1.294 | -0.158 | -0.002 | 0.789 | 0.656 | 2.72E-07 | **CLRN1-AS1** | **CLRN1 antisense RNA 1 (non-protein coding)** |
| chr6 | 41646751 | 41647250 | -1.342 | -0.013 | 0.017 | 0.393 | 0.246 | 3.17E-07 | TFEB | transcription factor EB (TFEB), transcript variant 1, |
| chr9 | 71412501 | 71413000 | -1.382 | 0.215 | 0.011 | 0.669 | -0.740 | 3.30E-07 | **PIP5K1B** | **phosphatidylinositol-4-phosphate 5-kinase,** |
| chr1 | 29137001 | 29137500 | 1.363 | -0.154 | -0.018 | -0.143 | 0.230 | 3.54E-07 | OPRD1 | opioid receptor, delta 1 (OPRD1), mRNA. |
| chr6 | 163684001 | 163684500 | 1.376 | 0.125 | 0.007 | -0.655 | 0.106 | 3.61E-07 | **PACRG** | **PARK2 co-regulated** |
| chr3 | 136715501 | 136716000 | 1.251 | -0.191 | 0.000 | -1.020 | 0.614 | 3.82E-07 | **IL20RB** | **interleukin 20 receptor beta (IL20RB), mRNA.** |
| chr6 | 96059251 | 96059750 | 1.346 | 0.332 | 0.002 | -0.305 | 0.074 | 4.64E-07 | MANEA | mannosidase, endo-alpha (MANEA), mRNA. |
| chr14 | 79080751 | 79081250 | 1.292 | 0.507 | 0.010 | 0.411 | 0.153 | 5.31E-07 | **NRXN3** | **neurexin 3 (NRXN3), transcript variant 1** |
| chr4 | 169542751 | 169543250 | 1.171 | 0.495 | -0.026 | -0.202 | 0.451 | 6.43E-07 | **PALLD** | **palladin, cytoskeletal associated protein** |
| chr14 | 90594001 | 90594500 | -1.160 | 0.189 | 0.020 | 0.883 | -0.633 | 6.69E-07 | **KCNK13** | **potassium channel, subfamily K,** |
| chr1 | 164845751 | 164846250 | 1.352 | -0.345 | 0.019 | -0.044 | -0.100 | 6.96E-07 | **PBX1** | **pre-B-cell leukemia homeobox 1** |
| chr6 | 87788251 | 87788750 | -1.254 | -0.261 | -0.011 | 0.276 | 0.474 | 7.02E-07 | 7SK | Rfam model RF00100 |
| chr6 | 35890501 | 35891000 | -1.228 | 0.341 | -0.028 | 0.565 | -0.404 | 7.46E-07 | SRPK1 | SRSF protein kinase 1 |
| Chr4 | 5298251 | 5298750 | 0.846 | 0.329 | -0.015 | 0.191 | -0.349 | 7.76E-07 | **STK32B** | **serine/threonine kinase 32B (STK32B), mRNA.** |
| chr6 | 51323001 | 51323500 | -1.298 | 0.128 | 0.010 | -0.300 | 0.168 | 7.86E-07 | SNORD66 | Rfam model RF00572 |
| chr4 | 53457501 | 53458000 | -1.307 | -0.181 | -0.019 | 0.025 | 0.450 | 8.29E-07 | **USP46** | **ubiquitin specific peptidase 46** |

Supplementary Figure 1: Averaged expression values of the ZBTB20 gene across ten brain regions


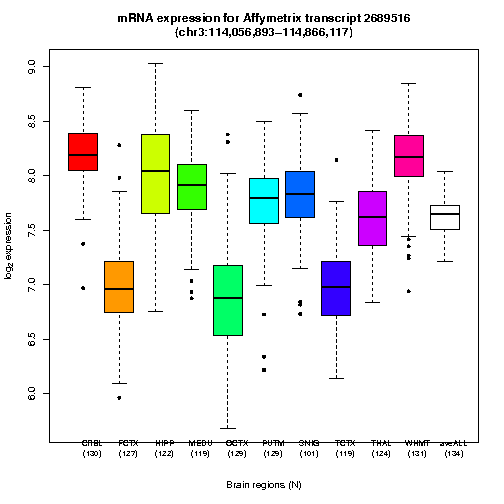


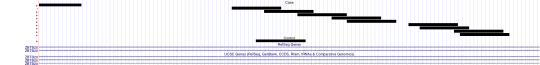


Supplementary Figure2. Example of discordant twin pair showing 10:1 ratio of case:control reads within the ZBTB20 DMR (chr3:114618751-114619251).


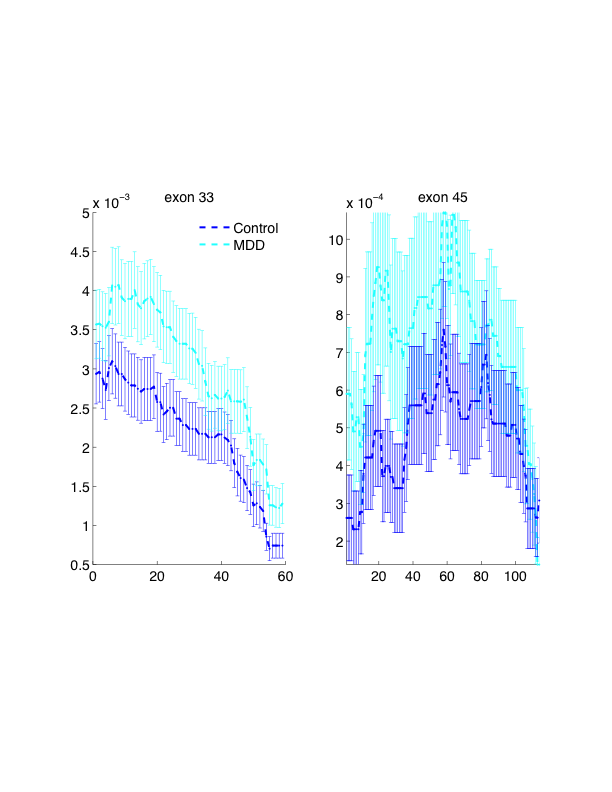


Supplementary Figure3. Average, per-base, normalized read counts in cases and controls for non-standard ZBTB20 exons 33 and 45 showing discordance for MDD. Positions are relative to the start of each exon. Error bars indicate the standard error.
